# Seasonal variation in the diagnosis of cancer: a study based on national cancer registration in Sweden

**DOI:** 10.1038/sj.bjc.6600901

**Published:** 2003-04-29

**Authors:** M Lambe, P Blomqvist, R Bellocco

**Affiliations:** 1 Department of Medical Epidemiology, Karolinska Institutet, PO Box 281, SE-171 77 Stockholm, Sweden

**Keywords:** neoplasms, detection, diagnosis, seasonal variation, malignant melanoma, breast cancer, prostate cancer, thyroid cancer

## Abstract

Data from the Swedish Cancer Register 1987–1996 were used to examine seasonal trends in the diagnosis of cancer. For melanomas, prostate, breast and thyroid cancer there were clear seasonal variations with reductions in the number of cases reported during the summer and December that are likely to reflect mainly hospital delays.

There are reports of seasonal variations in the diagnosis and reporting of malignant melanomas or pigmented naevi (Swerdlow, 1985; Schwartz *et al*, 1987; Braun *et al*, 1994), lymphomas (Westerbeek *et al*, 1998), leukaemias (Badrinath *et al*, 1997; Eatough, 2002), breast cancer (Cohen *et al*, 1983; Kirkham *et al*, 1985; Mason *et al*, 1990; Ross *et al*, 1997; Dimitrov *et al*, 1998; Gao *et al*, 2001), and childhood cancers (Ross *et al*, 1999). Some of these variations in presentation have intriguing temporal patterns which reflect either biological phenomena or administrative differences in the likelihood of tumour detection and registration. We judged that further exploration of this issue is of interest.

Data from a nationwide Cancer Register provided an opportunity to examine seasonal trends in the diagnosis of cancer in Sweden by analysing all invasive cancers recorded between 1987 and 1996, a total of more than half a million incident cases.

## MATERIAL AND METHODS

The Swedish Cancer Registry was established in 1958 and covers the whole Swedish population (8.9 million), including foreign citizens and ethnic minorities with permanent residency.

Reporting of all new cancers and also certain benign tumours is mandatory, both in public and private care. Separate notifications should be filed by the clinician as well as by the pathologist/morphologist, which means that at least two reports per case are needed before registration. Furthermore, a new report must be issued if a substantial revision of the original diagnosis is made. The completeness of recording is estimated to be 95%. Approximately 97% of all recorded cases are morphologically verified (Swedish Cancer Registry, 2000).

Since 1985, reports are first sent to one of six regional Cancer Registries where patient data and tumours are recorded. For each calendar year, the regional registries submit the material to the National Cancer Registry for collation, merging of data and updating of the national files. A coordination group with representatives from the National Registry and each regional registry meet regularly to ascertain conformity in coding and data-processing routines.

The Cancer Registry publishes an annual report with the numbers of cancers, gender and age-specific and age-standardised incidence rates. Complete cancer incidence data for each year are released with about 2 years delay. At present, approximately 45 000 incident cases are recorded annually. Date of diagnosis is defined as the earliest examination date when the cancer was found (Swedish Cancer Registry, 2000).

The present study analysed tumours reported as definitely malignant neoplasms, a total of 555 948 incident tumours during the 10-year period 1987–1996. No national information was available on stage.

## STATISTICAL ANALYSES

To detect any deviation from a uniform monthly distribution of the frequency of cancers by site throughout the year, a *χ*^2^ test for heterogeneity was used. In a subsequent approach, tumour reports from 1987 to 1996 were modelled by year and month to identify smooth trends during the years (Bacchetti, 1994). In our analysis, the temporal trend is a nuisance and was modelled initially both in continuous (linear and quadratic terms) and in a completely unstructured way using indicator variables. A better approach is to use parametric natural cubic B splines with evenly spaced knots (De Boor, 1978).

Monthly effects were modelled generally as *M*(*j*)=*M*(*j*+12) and *M*(1)=0 for 11 free parameters, or with the following reduced model:


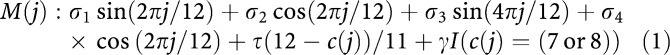


where *c*(*j*) is the calendar month (i.e., 1 if *j* is January of any year, 2 if *j* is February, etc.), the *σ*'s model any truly seasonal patterns, *τ* is the size of the jump between December and January and models any linear trend over the calendar year, and *γ* is a general summer effect (July and August) beyond that captured by the linear trend and the seasonal terms (the summer spike). The model uses only six parameters, instead of the 11 needed for completely arbitrary month effects, separating seasonal components from other influences.

Cancer incidence was modelled with both negative binomial regression to adjust for overdispersion and lack of fit in Poisson's regression. For each of the four types of cancers that deviated from a uniform temporal distribution, the parameter estimates were adjusted by gender, age and region. Model estimation and fitting was performed using STATA 7.0 (Stata Corp, 2000).

## RESULTS

We first examined the combined monthly distribution for all cancer sites (ICD-7: 140–209). Malignant melanomas, prostate, breast and thyroid cancers were the only sites for which a deviation from a uniform distribution throughout the year could be detected (Table 1

**Table 1:** Monthly frequency distributions of diagnosis of melanoma, breast, prostate and thyroid cancers in Sweden 1987–1996. *χ*^2^ test for heterogeneity

**Cancer**	**Jan**	**Feb**	**Mar**	**Apr**	**May**	**June**	**July**	**Aug**	**Sept**	**Oct**	**Nov**	**Dec**	**Mean**	**Total**	***χ*^2^ (d.f.)**	***P*-value**
Melanoma	1132	1136	1344	1246	1471	1652	1153	1362	1545	1502	1330	1098	1331	15971	277.7 (11)	<0.001
Breast	4812	5211	5618	5243	5293	5156	3480	3722	5211	5787	5804	4806	5012	60143	1181.4 (11)	<0.001
Prostate	5010	5209	5491	4843	5014	4407	3658	4244	5295	5704	5623	4644	4929	59142	825.8 (11)	<0.001
Thyroid	311	355	350	290	282	255	186	222	298	329	295	253	286	3433	98.3 (11)	<0.001

). For melanomas, the number of new cases reported was lowest during the winter months while monthly frequencies peaked at two different points: in May–June and September–October. For prostate, breast and thyroid, there was a clear decrease in the frequency of new cases diagnosed during the summer months, followed by a transient increase in subsequent months. The highest mean number of cases was reported in October and November for prostate and breast cancer, respectively. For all four cancer types, there was a decrease in December followed by an increase in January and February. Seasonal trends (based on 3-months moving averages) are illustrated in Figure 1

**Figure 1:**
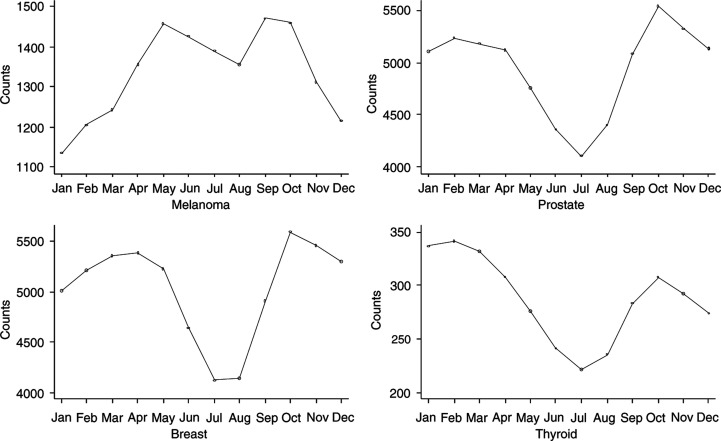
Number of new cancer cases (3-month moving averages) diagnosed in Sweden 1987–1996.

.

In a second analytic step, monthly counts from the whole period of observation were modelled in terms of secular trends and short-term effects to identify smooth trends over the year, adjusted by gender, age and region. Table 2

**Table 2:** Comparison of seasonal changes in cancer incidence estimated using model (1)

	**July and August decrease (*γ*)**		**January increase (*τ*)**	
**Type of cancer**	**Estimate**	**(s.e.)**	**Estimate**	**(s.e.)**
Melanoma	−28.4	4.7	10.6	7.6
Breast	−16.7	3.6	20.6	5.6
Prostate	−13.1	2.7	17.8	4.1
Thyroid	−27.2	9.2	27.6	9.2

The estimates for *γ* can be interpreted as the percentage decrease in July/August, and for *τ* as the percentage jump from December to January after controlling for seasonal effects and trend over calendar year.

shows a comparison of the relative seasonal changes in registration. The most pronounced summer season decrease was seen for melanomas (−28.4%), while thyroid cancer had the highest relative increase between December and January (+27.6%). When assessed by age at diagnosis, year of diagnosis, or by health-care region, the observed patterns did not change (data not shown).

## DISCUSSION

For the great majority of cancers, we found a uniform seasonal distribution of detection. However, for melanomas, prostate, breast and thyroid cancer there were reductions in the number of cases diagnosed during the summer and the month of December.

### Melanoma

For melanomas, our results broadly corroborate findings in at least three earlier studies that found evidence of seasonal patterns of detection (Swerdlow, 1985; Schwartz *et al*, 1987; Braun *et al*, 1994). At northern latitudes such as in Sweden, an early (May–June) peak could reflect an increased patient awareness and self-detection of abnormal naevi coinciding with the change to summer clothing. The postsummer (September–October) peak may, however, be a result of a promoting effect of sun exposure, resulting in signs and symptoms of malignant changes in pre-existing naevi.

### Breast cancer

The sharp decrease in the number of new cases of breast cancers recorded in June and July is likely to reflect mainly the reduced activity of mammography screening programmes during the summer months. These programmes were introduced in Sweden in the late 1980s. By 1993, more than 90% of women of eligible ages (generally 50–74 years) were invited to regional screening programmes. However, in a separate analyses of cases recorded during 1988 and earlier, a similar reduction during the summer was found. Patients' delay may also contribute to the summer decrease. Signs and symptoms are disregarded and not attended to until after vacation time. The results of earlier studies of possible seasonality of breast cancer have varied (Cohen *et al*, 1983; Kirkham *et al*, 1985; Mason *et al*, 1990; Ross *et al*, 1999; Gao *et al*, 2001), but none have found a similar pronounced summer decrease.

### Prostate and thyroid cancer

To our knowledge, no previous study has investigated seasonal variations in the diagnosis of prostate and thyroid cancer. As in the case of breast cancer, our findings of a decrease during the summer are likely to reflect a reduced capacity to ascertain suspected cases within the health-care system.

Our study is the largest reported on this subject so far. It was based on national cancer register data of high validity to which all tumours are reported in a country with a uniform national public health-care system. Given the consistency of our findings when assessed by region or time period, chance is an unlikely explanation for the observed patterns. It also appears unlikely that our results can be explained by systematically delayed reporting or data entry by season at the regional registries. If this had occurred, seasonal variations would have been observed for a broader range of cancers.

In conclusion, our findings of seasonal variations in the diagnosis of melanomas, thyroid, breast and prostate cancer are likely to reflect both patient and hospital delay. A common clinical feature of these four cancers is that they often present with mild, nonacute symptoms, allowing delay of self-referral. It is also conceivable that the lower diagnostic intensity during summer and around the turn of the year reflects a reduced capacity to evaluate patients with suspected cancers. For melanomas, however, the bimodal diagnostic pattern would be in keeping with seasonal exposure to solar radiation.

Overall, the observed variations in detection intensity appear unlikely to have clinical implications or influence survival negatively, since very rapid progression is rare in these tumours. However, any seasonally related delay between first visit, referral and final diagnosis of cancer because of a reduced capacity within the health-care system may cause anxiety among patients. Further efforts to minimise delays are important, for example, by implementing quality guidelines where patients with a suspected cancer are guaranteed to be evaluated, diagnosed and treated within a stipulated time period.
